# Mild Encephalopathy/Encephalitis With a Reversible Splenial Lesion (MERS) and Longitudinally Extensive Transverse Myelitis (LETM) in Influenza B: Neurotropic Mechanisms and Diagnostic Challenges

**DOI:** 10.7759/cureus.30681

**Published:** 2022-10-25

**Authors:** Bahadar S Srichawla

**Affiliations:** 1 Department of Neurology, University of Massachusetts Chan Medical School, Worcester, USA

**Keywords:** neurotropism, splenium, mild encephalopathy/encephalitis with reversible splenial lesion, neuroinfectious diseases, corpus callosum, influenza virus, influenza b, encephalomyelitis, longitudinally extensive transverse myelitis, infectious encephalitis

## Abstract

Mild encephalopathy/encephalitis with a reversible splenial lesion (MERS) and longitudinally extensive transverse myelitis (LETM) are neuroinflammatory conditions related to the brain and spinal cord, respectively. Most cases of MERS and LETM are related to a secondary autoimmune process in response to an initial insult (i.e., infection, immunization, etc.). The case of an 18-year-old female who developed a three-day history of fever, quadriplegia, cough, and mild encephalopathy is reported here. The patient tested positive for influenza B by nasopharyngeal swab with polymerase chain reaction (PCR). Initial magnetic resonance imaging (MRI) revealed the presence of a diffusion-restricted non-enhancing lesion confined to the splenium of the corpus callosum (MERS type I) and longitudinally extensive non-enhancing T2 hyperintensities from C1 to C5. The patient was managed with a five-day course of 1,000 mg of intravenous methylprednisolone (IVMP). Additionally, five days of therapeutic plasmapheresis (PLEX) was completed. The patient showed significant improvement with medical management and physical therapy. At the one-year follow-up, her motor symptoms had resolved and endorsed only mild paresthesia in the upper extremities. A repeat MRI revealed a reversal of the splenium lesion and moderate improvement in T2 hyperintensities of the cervical cord. Assessing neuroinvasion of the influenza virus is difficult, and diagnostic challenges arise in determining primary infectious versus autoimmune-mediated neuroinflammation. A review of the literature on influenza infection with radiographic findings of MERS and LETM is included.

## Introduction

Influenza B is a negative single-stranded RNA virus that belongs to the Orthomyxoviridae family. Both influenza A and B are primarily viral respiratory infections; however, cases of neurological sequelae secondary to influenza have been reported. This is of particular concern given the annual seasonal spike of cases seen around the world from these viruses. Altered mental status has been commonly reported and is termed influenza-associated encephalopathy (IAE). Cases of IAE have been reported more frequently in children. Although cases of encephalopathy have been reported, influenza is not considered a virus with considerable neuroinvasive potential. In fact, only a few cases of IAE with the detection of influenza RNA in cerebrospinal fluid (CSF) have been reported [1].

Mild encephalitis with a reversible splenial lesion (MERS) is a clinical radiographic syndrome defined by encephalopathy and a reversible lesion in the splenium of the corpus callosum. Typically, cases have been reported from East Asia and Japan. Here, a case is reported from the United States. Longitudinally extensive transverse myelitis (LETM) is defined as a longitudinal lesion that occurs at three or more consecutive spinal levels. LETM has been associated with demyelinating diseases such as neuromyelitis optica spectrum disorder (NMOSD) and myelin oligodendrocyte glycoprotein-associated disease (MOGAD). Clinical etiology also includes bacterial, fungus, and viral myelitis.

The case of an 18-year-old female with clinical and radiographic findings consistent with encephalomyelitis is reported. Lesions consistent with mild encephalitis with a reversible splenial lesion (MERS) and longitudinally extensive transverse myelitis (LETM) from C1 to C5 are rarely observed together [2].

## Case presentation

**Figure 1:**
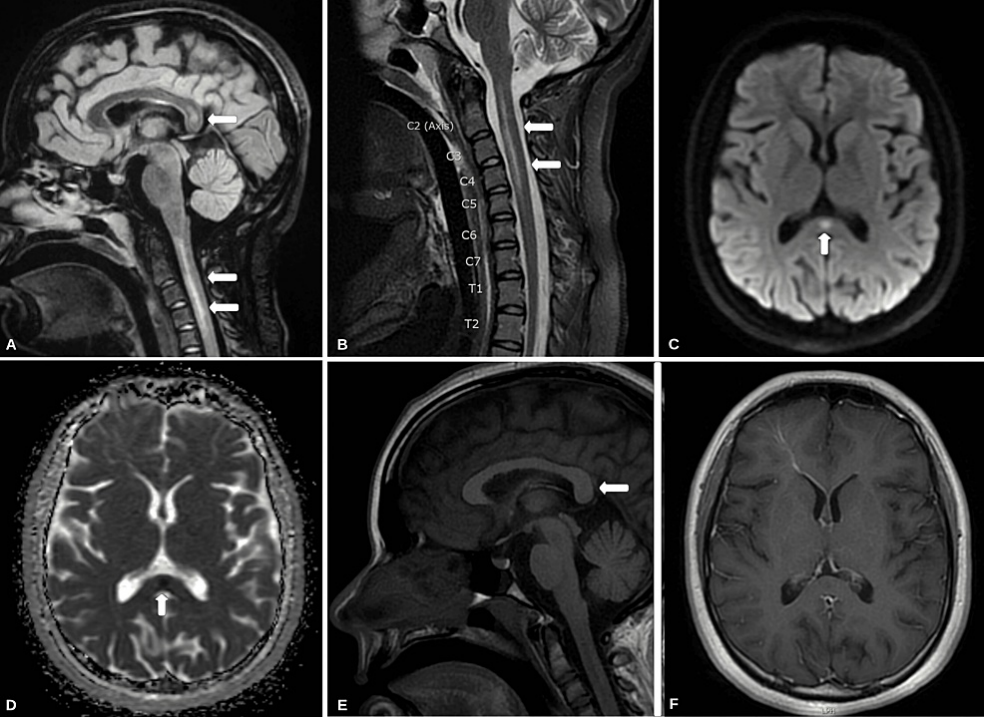
MRI of the brain and cervical spinal cord (A) T2 FLAIR sagittal sequence shows a non-enhancing lesion in the splenium of the corpus callosum (arrows). (B) T2 FLAIR sagittal sequence of the cervical spinal cord with hyperintensities from C1 to C5 (arrows). (C) DWI sequence depicts diffusion restriction within the splenium of the corpus callosum (arrow). (D) ADC sequence confirming the aforementioned diffusion restriction (arrow). (E and F) T1-weighted imaging with only mild hypointensity in the splenium of the corpus callosum (arrow) and without enhancement. MRI: magnetic resonance imaging, FLAIR: fluid-attenuated inversion recovery, DWI: diffusion-weighted imaging, ADC: apparent diffusion coefficient

**Figure 2:**
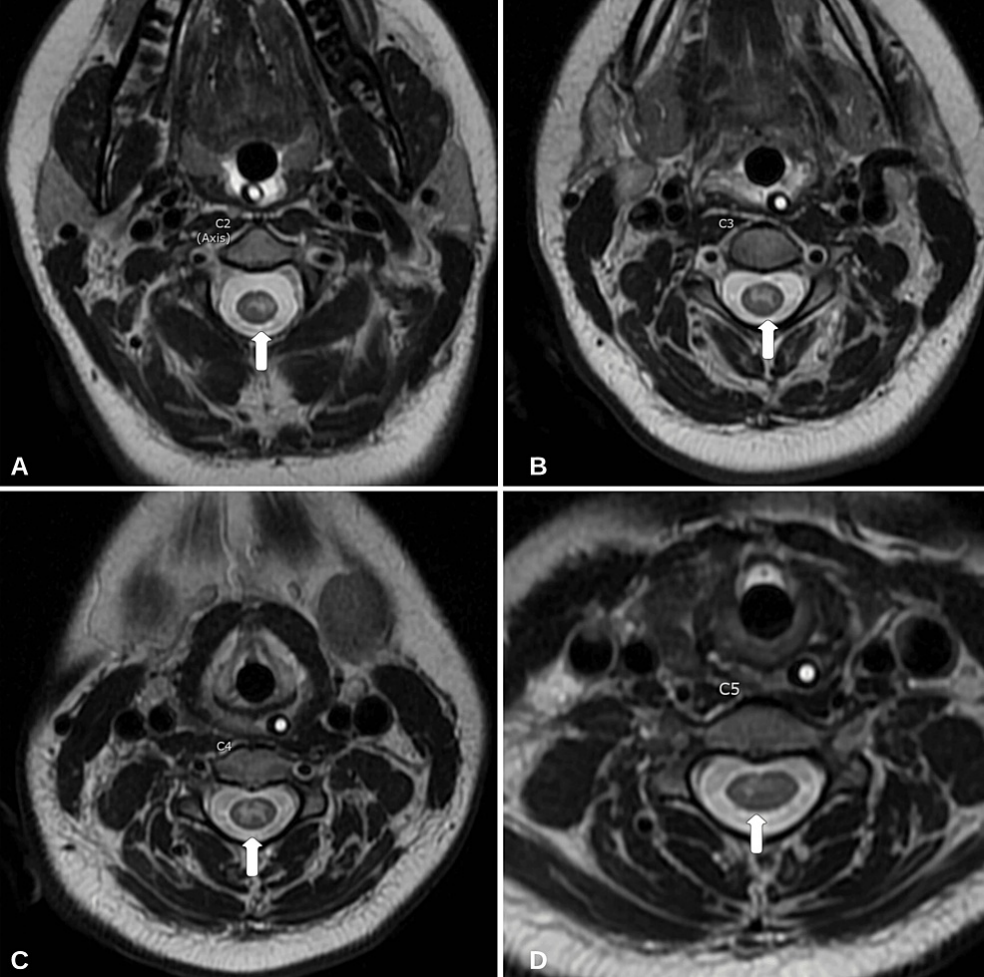
MRI (axial view) of the cervical spine (A-D) T2 FLAIR (axial view) of spinal segments C2-C5 reveals primarily gray matter hyperintensities (arrows). MRI: magnetic resonance imaging, FLAIR: fluid-attenuated inversion recovery

An 18-year-old right-handed female presented during the northern hemisphere influenza season (October-April) with a three-day history of progressively worsening cough, dyspnea, and weakness. Prior medical and family history was unremarkable. Vital signs at presentation were significant for a temperature of 38.9°C. Initial neurological examination was significant for profuse muscle weakness. The Medical Research Council (MRC) Scale for Muscle Strength grading (0-5) revealed 1/5 muscle strength in the bilateral lower extremities both proximally and distally. The patient was unable to maintain neck flexion against gravity. Muscle strength in the upper extremities was 3/5. Reflexes were 3+ on the bilateral patella and Achilles tendon, with two beats of clonus, a mute Babinski reflex, and a positive Hoffman reflex on the right. All other reflexes were 2+ and symmetric. She was oriented to person, place, and time and exhibited some memory loss consistent with mild encephalopathy. The rectal tone was normal, and she was continent of urine. Formal neurocognitive testing was not completed on the initial presentation. A chest radiograph demonstrated hypoinflated lungs. The complete blood count and comprehensive metabolic panel revealed no leukocytosis or lactic acidosis. The patient began to develop progressive bradypnea and was subsequently intubated and brought to the intensive care unit (ICU) for further treatment. She tested positive for influenza B through polymerase chain reaction (PCR) cultured from a nasopharyngeal swab. A magnetic resonance imaging (MRI) of the brain with and without gadolinium contrast (Gd+) revealed an isolated 11-mm diffusion-restricting lesion within the splenium of the corpus callosum that is hyperintense on T2-weighted imaging (T2W1) and hypointense on T1-weighted imaging (T1WI) (Figure 1). An MRI of the cervical spine with and without Gd+ showed a predominantly gray matter lesion from C1 to C5 segments of the cervical spinal cord consistent with longitudinally extensive transverse myelitis (LETM) (Figure 2). None of the identified lesions were contrast-enhancing.

**Figure 3:**
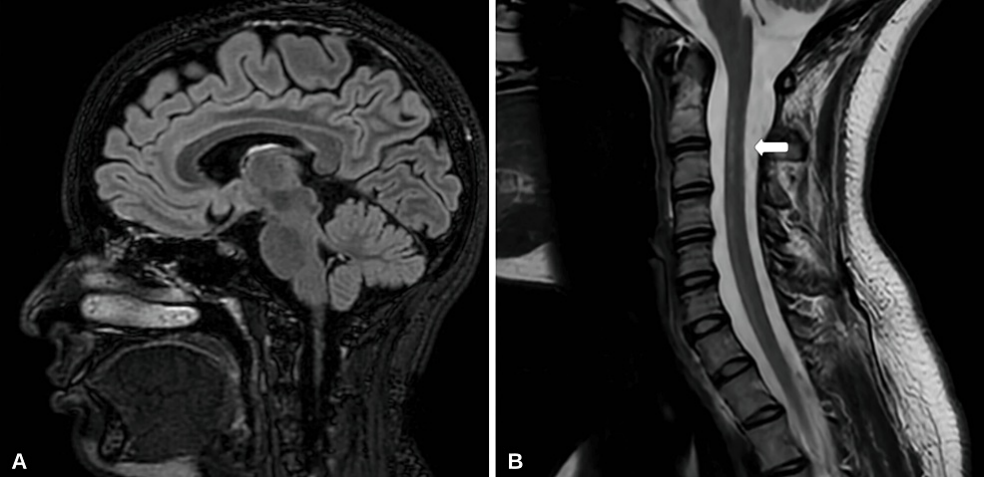
Repeat MRI of the brain and cervical spine (A) MRI of the brain T2/FLAIR sagittal sequence reveals resolution of previous splenium lesion on the corpus callosum. (B) MRI of the cervical spine T2/FLAIR sagittal sequence depicts improvement in hyperintense lesions (arrow). MRI: magnetic resonance imaging, FLAIR: fluid-attenuated inversion recovery

Cerebrospinal fluid (CSF) analysis for influenza B was negative. CSF chemistry revealed an albumin level of 9 mg/dL (normal range: 0-35 mg/dL), glucose of 66 mg/dL (normal range: 50-80 mg/dL), and immunoglobulin G (IgG) of 1.4 mg/dL (normal range: 0-6 mg/dL). CSF analysis for oligoclonal bands, aquaporin-4 (AQP4) antibody/neuromyelitis optica (NMO), and myelin oligodendrocyte glycoprotein (MOG) antibody was also negative. Serum evaluation for NMO, MOG, and autoimmune panel was also negative. The serum IgG level was elevated at 1,760 mg/dL (normal range: 768-1,632 mg/dL). Concerned about an infectious or para-autoimmune process, high-dose intravenous methylprednisolone (IVMP) was administered at 1,000 mg for five days. Due to difficulties with weaning from mechanical ventilation, five days of therapeutic plasmapheresis (PLEX) was completed after IVMP therapy. Extubation was achieved on the 10th day of admission, and moderate improvement in muscle weakness was observed. On the 15th day of admission, the patient was discharged to a rehabilitation facility and had achieved 4/5 muscle strength in her lower extremities. Upper extremity strength and neck flexion were 5/5. After completing three weeks of intensive inpatient rehabilitation, the patient was sent home but still required a cane for assistance with walking. At the one-year follow-up appointment, she endorsed only mild upper extremity paresthesia and had regained full motor strength. A repeat MRI of the brain and cervical spine was performed (Figure 3).

## Discussion

Mild encephalitis/encephalopathy with a reversible splenial lesion (MERS), not to be confused with the Middle Eastern respiratory virus, is a clinical radiographic syndrome characterized by the presence of mild encephalopathy and a transient lesion within the splenium of the corpus callosum (MERS type I) or frontal white matter (MERS type II). The condition is more prevalent among children and less often reported in adults. The isolated splenial lesion is hypothesized to be secondary to rapid cytotoxic and intramyelinic edema secondary to neuroinflammation that resolves. The reported etiologies of MERS include metabolic abnormalities, infection, autoimmune conditions, toxins, and drug withdrawal [3,4]. The case reported here is that of MERS secondary to acute influenza infection. The patient had a significant improvement in her symptoms with high-dose IVMP and therapeutic plasmapheresis. The resolution of the splenium lesion on subsequent MRI and the resolution of mild encephalopathy are consistent with this diagnosis. Acute disseminated encephalomyelitis (ADEM) can be considered a differential diagnosis of MERS. However, ADEM typically presents with gadolinium enhancement (depending on the acuity) and without diffusion restriction. Furthermore, MRI findings of ADEM include asymmetric white matter disease less often involving the corpus callosum. In this case, the presence of an isolated non-enhancing diffusion-restricted lesion within the splenium of the corpus callosum points away from ADEM and toward MERS [5].

Longitudinally extensive transverse myelitis (LETM) is defined as a longitudinal lesion that extends through three or more spinal segments. LETM is typically associated with neuromyelitis optica spectrum disorder (NMOSD) and myelin oligodendrocyte glycoprotein-associated disease (MOGAD), where the latter condition has a more favorable outcome. The diagnosis of LETM is supported by the presence of bilateral symptoms, evidence of active neuroinflammation (i.e., pleocytosis, high IgG index, etc.), sensory level of the trunk, and the lack of a more appropriate diagnosis [2]. In the presented case, bilateral symptoms with weakness of the lower extremities and hyperreflexia were observed. The diagnosis was supported by a para-infectious process of an active influenza B infection.

**Figure 4:**
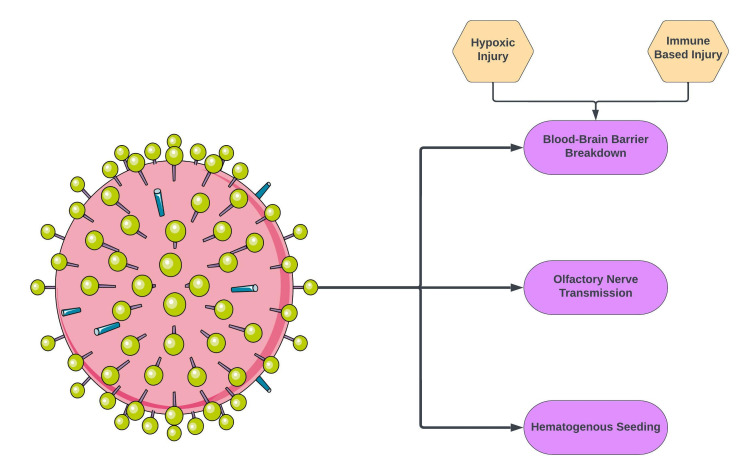
Proposed mechanisms of viral neurotropism Image credits: Bahadar S. Srichawla

**Figure 5:**
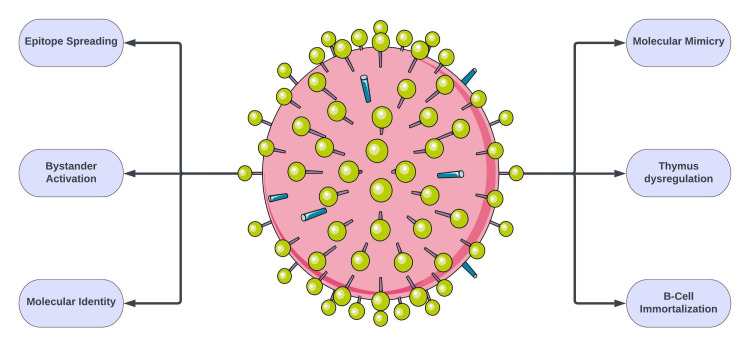
Proposed mechanisms of viral autoimmunity Image credits: Bahadar S. Srichawla

Infection of nervous tissue is observed in neurotropic viruses such as rabies, herpes simplex, and poliovirus. Radiographic findings of these viruses can vary. One study across two influenza seasons identified nine patients with influenza-associated encephalopathy (IAE). No evidence of influenza neuroinvasion was observed. Similarly, in this reported case, significant central nervous system (CNS) disease is clearly observed; however, neuroinvasion through influenza B CSF analysis is non-contributory [6]. One hypothesis is that active infection leads to overstimulation of the immune system and causes secondary neuroinflammation. Such events have been recorded in the past with immunization-related transverse myelitis and encephalitis. Furthermore, in this reported case, the utilization of known immunomodulators such as high-dose IVMP and PLEX led to improvement in symptoms. The lack of evidence on CSF is not limited only to the influenza virus. There have been multiple instances of significant CNS disease from a virus without direct neuroinvasive CSF evidence (i.e., monkeypox virus and coronavirus) [7]. The mechanisms by which viruses exert neurotropism include blood-brain barrier (BBB) breakdown, retrograde axonal transport from a peripheral nerve, and direct hematogenous seeding (Figure 4) [8]. The proposed mechanisms of autoimmunity include molecular mimicry, bystander activation, and B-cell immortalization, among others (Figure 5) [9].

Literature review

A literature search was conducted utilizing PubMed/PubMed Central/Medical Literature Analysis and Retrieval System Online (MEDLINE) database to identify similar cases of influenza-mediated encephalitis and myelitis. Records identifying mild encephalitis with a reversible splenial lesion (MERS) and longitudinally extensive transverse myelitis (LETM) were included. A gray literature search was conducted reviewing the first 100 results of Google Scholar. Backward and forward citation was utilized to further expand the literature search. Records without the full text available, non-English, and not peer-reviewed were excluded. Data extracted includes reference, study type, sample size, patient age, gender, clinical symptoms, neuroimaging, diagnostic modality, management, and outcomes. A total of eight records are identified and included in Table 1.

**Table 1:** Records identified from literature search F: female, M: male, MRI: magnetic resonance imaging, PCR: polymerase chain reaction, LETM: longitudinally extensive transverse myelitis, MERS: mild encephalitis with a reversible splenial lesion, Gd+: gadolinium enhancement, AQP: aquaporin-4 antibody, NMO: neuromyelitis optica, T2WI: T2-weighted imaging, T1WI: T1-weighted imaging, IVIG: intravenous immunoglobulin, IVMP: intravenous methylprednisolone, MOG: myelin oligodendrocyte glycoprotein, MOGAD: myelin oligodendrocyte glycoprotein-associated disease, ANA: antinuclear antibody

Reference	Study type	Extracted sample size	Age	Gender	Clinical symptom(s)	Diagnostics	Neuroimaging	Diagnosis	Management	Outcomes
Ka et al. (2015) [10]	Case series	1	5	F	Fever, lethargy, ataxia, mildly decreased consciousness	Influenza B via PCR	MRI: focal diffusion restriction in the corpus callosum	Influenza B MERS	Supportive	Recovery within 72 hours, discharged on day 5
Takahashi et al. (2020) [11]	Case report	1	50	M	Fever, altered mental status, incoherent speech, upper extremity myoclonus, + Kernig sign	Influenza B via rapid antigen test	MRI: focal diffusion restriction within the splenium of the corpus callosum	Influenza B MERS	Supportive	Resolution
Takatsu et al. (2017) [12]	Case report	1	31	F	Mild encephalopathy, dysosmia	Influenza A via rapid antigen test	MRI: T2WI hyperintensity with diffusion restriction and T1WI hypointensity within the splenium of the corpus callosum	Influenza A MERS	Oseltamivir and supportive	Resolution of splenium lesion by day 14, full recovery
Imataka et al. (2020) [13]	Case report	1	6	F	Encephalopathy	Influenza A via rapid antigen test	MRI: T2WI hyperintensity with diffusion restriction and T1WI hypointensity within the splenium of the corpus callosum	Influenza A MERS	High-dose IVMP, IVIG	Recovery
Fluss et al. (2010) [14]	Case report	1	2	F	Irritable, absent speech, truncal ataxia	Influenza A via PCR	MRI: T2WI hyperintensity with diffusion restriction and T1WI hypointensity within the splenium of the corpus callosum	Influenza A MERS	Supportive	Recovery
Amano et al. (2014) [15]	Case report	1	32	M	Fever, dysesthesia, lower extremity muscle weakness, urinary retention	Influenza A via PCR, ANA titer 1:320, MOG titer 65,536	MRI: non-enhancing T2WI hyperintensities from C2 to conus	Influenza A-positive MOGAD LETM	High-dose IVMP for five days	MOG titers decreased to 16,384 on day 7 and decreased to 4,096 on day 30
Márquez et al. (2022) [16]	Case report	1	30	F	Urinary retention, lower extremity paraparesis, upper extremity weakness, optic nerve and macular edema	Influenza A rapid antigen test	MRI: T2WI hyperintensities from C4 to T12	Influenza A LETM	High-dose IVMP for three days and six cycles of monthly intravenous cyclophosphamide	Recovery
Nakamura et al. (2011) [17]	Case report	1	15	F	Visual disturbance, dysuria, dysesthesia, hyperreflexia in the lower extremities	Influenza A via PCR, AQP4/NMO negative	MRI: T2WI hyperintensities from T2 to T5, Gd+ enhancing lesion in the right parietal lobe and hyperintense lesion in the left occipital and parietal white matter	Influenza A-associated NMO LETM	High-dose IVMP for three days	Recovery

Of the eight identified patients, 6/8 (75%) are identified as females. The average age is 21.3 years. Of the cases, 4/8 (50%) are documented in the pediatric population (<18 years of age). Five of the eight identified patients (62.5%) of the reported cases are that of MERS and three (37.5%) of LETM. Influenza A was diagnosed in 6/8 (75%) of cases and influenza B in 2/8 (25%). Amano et al. reported the case of a 32-year-old male with fever, weakness of the lower extremities, and urinary retention that was positive for influenza A by PCR. Antinuclear antibody (ANA) and MOG antibody titers were significantly elevated at 1:320 and 65,536, respectively [15]. This case points to an autoimmune condition that developed secondary to influenza A infection rather than direct neuroinflammation. The patient reported by Márquez et al. had a concurrent history of systemic lupus erythematosus [16]. The case of Nakamura et al. had negative serum tests for AQP4/NMO antibodies, but the diagnosis was made based on revised criteria [17].

Edet et al. also reported the case of a 32-year-old male presenting with a subacute onset of cough and confusion. He was diagnosed with an acute influenza A infection, and neuroimaging was consistent with significant periventricular hyperintensities with a diffusion-restricting lesion in the corpus callosum. The diagnosis of IAE was made, and radiographic findings were more closely associated with ADEM. Despite aggressive treatment, the patient could not be weaned from mechanical ventilation and was subsequently terminally extubated [18].

## Conclusions

The case of an 18-year-old female diagnosed with influenza B virus infection with clinical and radiographic findings of encephalomyelitis was discussed. This is the first reported case of influenza B with findings consistent with both mild encephalitis with a reversible splenial lesion (MERS) and longitudinally extensive transverse myelitis (LETM). Determining viral neurotropism remains difficult with negative CSF findings. Neuroinflammation from direct infectious involvement or secondary autoimmune reaction is discussed. A literature review on cases of influenza-associated MERS and LETM is included.
